# Broadband microwave coding metamaterial absorbers

**DOI:** 10.1038/s41598-020-58774-1

**Published:** 2020-02-04

**Authors:** Manh Cuong Tran, Van Hai Pham, Tuan Hung Ho, Thi Thuy Nguyen, Hoang Tung Do, Xuan Khuyen Bui, Son Tung Bui, Dac Tuyen Le, The Linh Pham, Dinh Lam Vu

**Affiliations:** 1 Faculty of Physics, Hanoi National University of Education, 136 Xuan Thuy, Cau Giay, Hanoi, Vietnam; 2 Institute of Physics, Vietnam Academy of Science and Technology, 18 Hoang Quoc Viet, Hanoi, Vietnam; 3 Institute of Materials Science, Vietnam Academy of Science and Technology, 18 Hoang Quoc Viet, Hanoi, Vietnam; 4 Department of Physics, Hanoi University of Mining and Geology, 18 Pho Vien, Bac Tu Liem, Hanoi, Vietnam; 5 Graduate University of Science and Technology, Vietnam Academy of Science and Technology, 18 Hoang Quoc Viet, Hanoi, Vietnam

**Keywords:** Materials science, Optics and photonics, Physics

## Abstract

In this paper, a broadband metamaterial microwave absorber is designed, simulated and measured. Differently from the traditional method which is only based on unit cell boundary conditions, we carried out full-wave finite integration simulations using full-sized configurations. Starting from an elementary unit cell structure, four kinds of coding metamaterial blocks, 2 × 2, 3 × 3, 4 × 4 and 6 × 6 blocks were optimized and then used as building blocks (meta-block) for the construction of numerous 12 × 12 topologies with a realistic size scale. We found the broadband absorption response in the frequency range 16 GHz to 33 GHz, in good agreement with the equivalent medium theory prediction and experimental observation. Considering various applications of metamaterials or metamaterial absorbers in the electromagnetic wave processing, including the radars or satellite communications, requires the frequency in the range up to 40 GHz. Our study could be useful to guide experimental work. Furthermore, compared to the straightforward approach that represents the metamaterials configurations as 12 × 12 matrices of random binary bits (0 and 1), our new approach achieves significant gains in the broadband absorption. Our method also may be applied to the full-sized structures with arbitrary dimensions, and thus provide a useful tool in the design of metamaterials with specific desired frequency ranges.

## Introduction

Research involving metamaterial has been massively focused by many groups worldwide in recent decades^1^. Metamaterial is an artificially structured material, generally made up of periodic metal circuits on a dielectric substrate. This material was first theoretically predicted by Veselago in his paper on negative refractive index^2^. But, almost four decades later, based on experimental evidence, the importance and applicability of metamaterial have been confirmed^3,4^. This material has a variety of electromagnetic and optical properties different from ordinary materials, leading to many special applications such as super-lens, cloaking, wireless power transfer, high-performance antenna systems^1,4,5^, and most recently the electromagnetic perfect absorber that the energy of the incident wave is mostly absorbed^2,6,7^. This dernier special feature allows many potential applications such as increased solar cell efficiency, military radar camouflage, super-sensitive sensor design and so on to be realized^8–18^. For achieving efficient applications, current research focuses on two main issues: controlling the absorption band and finding ways to extend the working frequency range. Different studies have been carried out for perfect absorber in the frequency domain from microwave to visible^18–34^. Various methods have been proposed and yielded positive results such as multilayered, asymmetric, super-cell structure, all-metal or all-dielectric structure, and hybrid structure^34–46^. Even now research has been geared up towards digital or coding metamaterial absorbers, which is related to computational metamaterial, to increase the applicability and meet the development of digital technology^47–59^. However, the challenge is that experimental structures often have high complexity, low flexibility, and therefore cause limitations in application.

Next, the study of the metamaterial absorber is performed generally from one unit cell with appropriate boundary conditions in the simulation, where the structure is assumed to be infinite. With such a simulation model, controlled defects in the structure due to its periodicity cannot be investigated. In this study, we consider a full-sized structure, which resembles the structure in a realistic size. Therefore, the introduction of controlled defects for absorption manipulation is feasible and realistic.

Studying the application of defects in the full-sized absorber for broadening the working frequency band has been described in our previous reports^30,31^. It can be seen that the method has many advantages such as simple structure, high flexibility, easy to integrate into the electronic or optical circuits. The challenge to this method is that the optimization process is quite complex and takes a lot of work. This work integrates the digital concept into the metamaterial absorber system, namely coding metamaterials. Coding and digital metamaterials are a new field of research and attract a lot of attention^48,49^. Researches often focus on investigating the ability to control the characteristics of reflected beams with different coding configurations^53–59^. Here, in this study we focus on investigating the electromagnetic wave absorption performance of the structure with different coding metasurfaces through the reflection and transmission parameters. Furthermore, in this study we place more emphasis on the ability to improve the performance of the meta-structure through a search algorithm using a large number of different coding configurations.

For the calculation of the absorption, 200 samples are used in each approach, we obtain the optimal configuration with a broadband up to 17 GHz, the results then theoretically analyzed and experimentally confirmed. This approach is expected opening up a new way to study and design broadband or multiband absorber metamaterial. Moreover, this method promises to fit very well with studies of the actual size structure, which requiring pre-analysis of electromagnetic response via simulation.

## Full-sized Absorber Design

**Figure 1:**
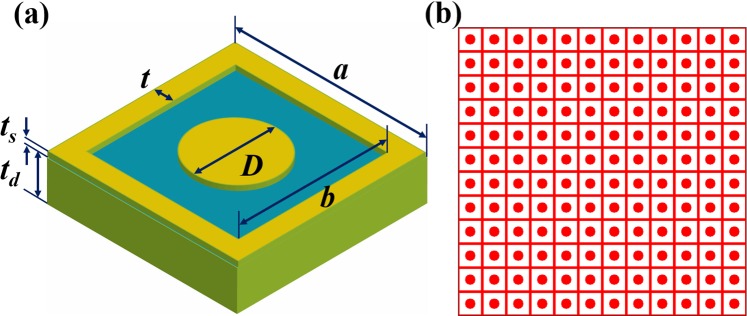
(**a**) An elementary unit cell with its dimension, (**b**) A full-sized structure of 144 UCs.

The basic unit cell (UC) is applied to generating the full-sized configurations. Figure 1 depicts a UC with its dimension and a full-sized structure of 144 UCs (12 × 12 UCs). For the top layer (copper) of a unit cell, the dimensions of the porous square are *a* = 9 mm; *b* = 7.8 mm, *t* = 0.6 mm, the thickness *t*_*s*_ = 0.03 mm, the diameter of the circle dish is *D = *3.5 mm, the electric conductivity of copper is σ = 5.82 × 10^7^ Sm^-1^. A dielectric substrate (FR4) with a dielectric constant of 4.3 and a loss-tangent of 0.025 is used in the middle layer. The thickness of the substrate (*t*_*d*_) is 1.5 mm. A full copper film (thickness *t*_*s*_) covers the bottom layer. We chose such a unit cell structure because of its simplicity and the ease of control to the desired working frequency range by rescaling to its dimensions^6^.

We use CST Microwave Studio which based on the Finite Integration Technique (FIT) for the simulation of wave-matter interaction. The open boundary conditions are applied in this study. In the simulation, a waveguide port was placed in front of the configuration and the electromagnetic wave is propagated at normal incidence to the surface, the (E, H) plane is parallel to the structure. The absorption spectra can be calculated as A(ω) = 1 – R(ω), where R(ω) is reflection spectra (the transmission is zero due to the thick bottom copper layer).

### Combination process

In the first approach, named SRS (simple random sample) method, to ensure the rationality of the full-sized structure and adapt to our calculation system, the configuration of 144 UCs (12 × 12 UCs) is investigated. The idea is that the defect positions or bits 0 will be generated by randomly removing the top copper layer of the UCs at an arbitrary position on the configuration, the locations with a normal UC are assigned as bits 1. In principle, the structure of *n* × *n* UCs could generate 2^n×n^ configurations. The structure of 144 UCs can generate therefore 2^144^ configurations and the possible number of simulations is very large. Each generated configuration will be transferred to the environment with appropriate boundary conditions to simulate the electromagnetic absorption response. The final results are aggregated and analyzed to provide optimal configuration.

**Figure 2:**
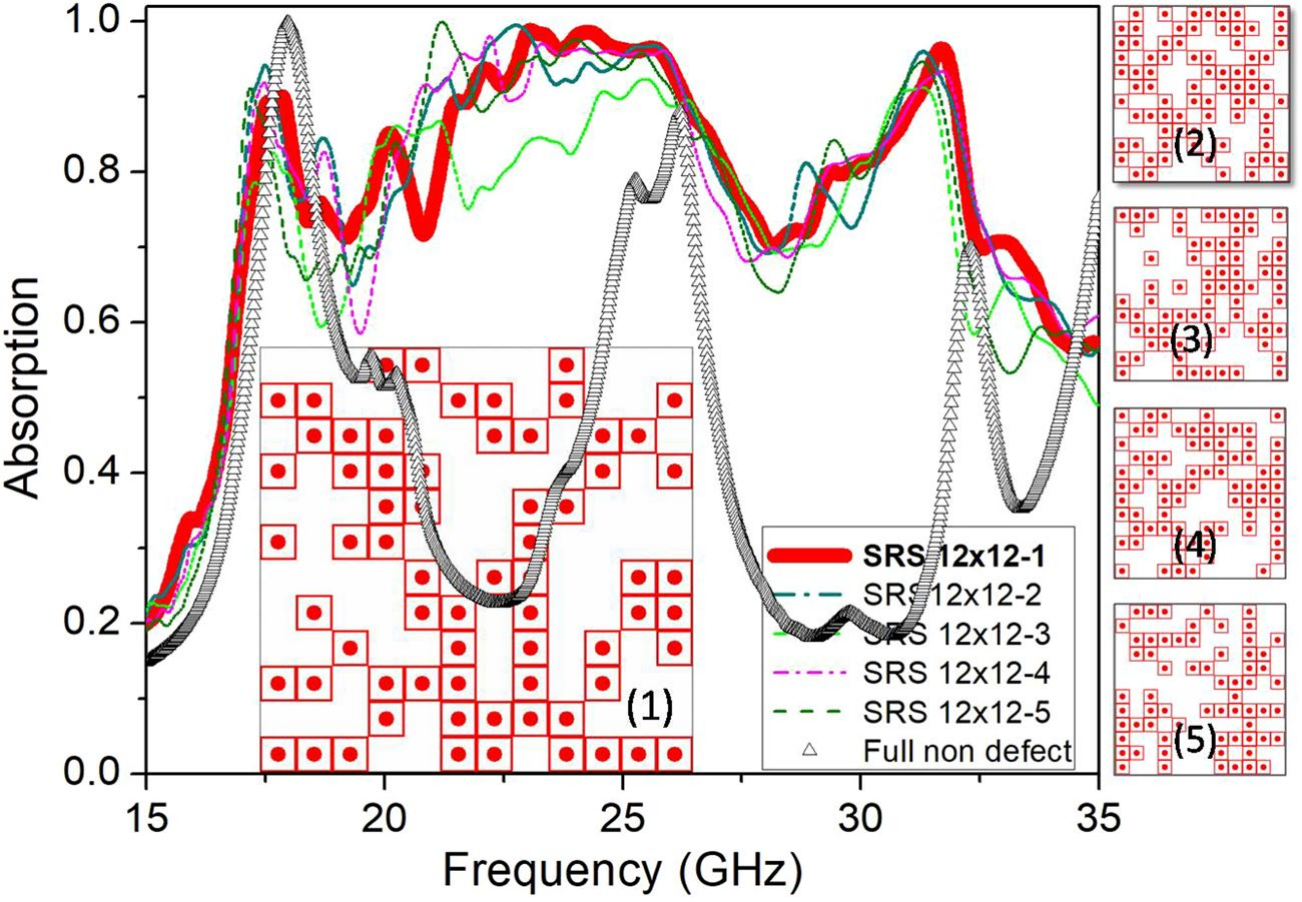
Absorption spectrum of SRS configurations and of the full-sized structure of 144 UCs. Right panels are different configurations SRS 12 × 12–2 to SRS 12 × 12–5.

It is worth mentioning that a simple random sample is a subset of a statistical population in which each member of the subset has an equal probability of being chosen. As an example, we draw multiple samples consisting of 200 configurations each from a set of possible 2^144^ configuration (configurational space). Here, the variable *s* is used to represent the size of the sample; thus configuration space size *N* = 2^144^ and sample size *s* = 200). By randomizing the selection procedure, any member of this configuration space has an equal chance of being selected as part of this first sample, and an equal chance of being selected for the next sample of the same size (and so on). After the simulation process, the absorption spectra of 5 selective cases (SRS 12 × 12–1 to SRS 12 × 12–5) are shown for a convenience view in Fig. 2.

One can see that a wide absorption band of ~17 GHz from 16 to 33 GHz (with the average absorptivity is about 85%) is obtained for almost of cases. We also present the absorption of the non-defect full-sized absorber with 144 UCs for the comparison (Fig. 2). The absorption range is improved for the case of applying defect generation algorithms in the structure.

This is a favorable result and can be used to confirm that by randomly generate the defects on the structure, a remarkable broad absorption frequency band can be obtained.

However, it is easy to point out that the absorption band for most random configurations has a deep recess around the 19 GHz and 28 GHz region. This suggests us to find out a more optimal strategy that can improve the absorption strength in the range. The next section will discuss this.

The second approach is named CFM (combination of fundamental meta-block), based on the combination process of the basic meta-blocks (MBs). MB is a set of basic blocks with the number of UC greater than 1, in this study, MBs are created from blocks including 2 × 2, 3 × 3, 4 × 4 and 6 × 6 UCs, which are the divisors of 12. Derived from the idea that relevant absorption peaks can overlap and form a broader absorption band, first we calculate the absorption of the different MBs, then the structures can combine to have the broadband selected to build a full-sized structure (of 144 UCs).

**Figure 3:**
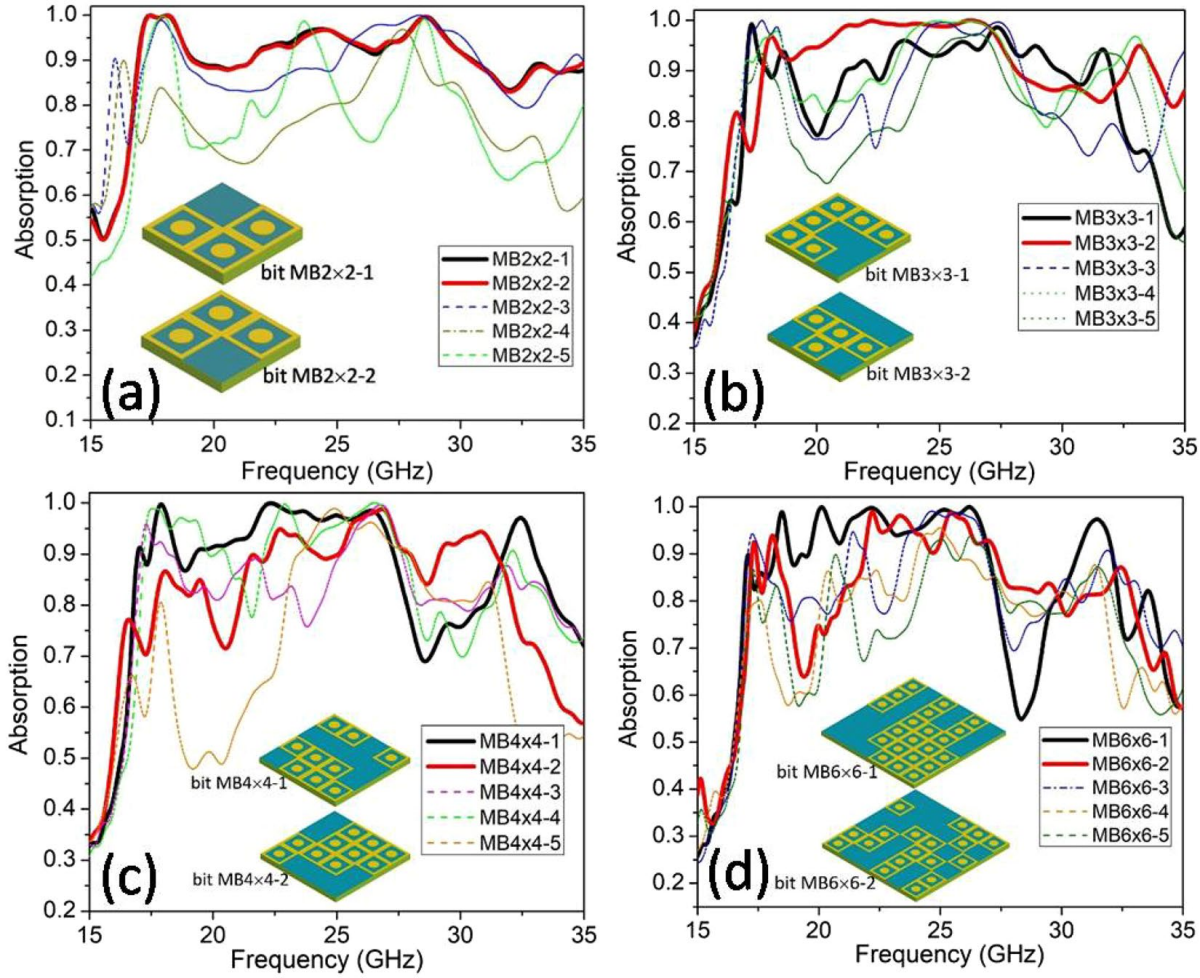
Absorption spectrum of different fundamental MB types: (**a**) MB2 × 2, (**b**) MB3 × 3, (**c**) MB4 × 4 and (**d**) MB6 × 6.

The first case discussed is MB of 4 UCs (called MB2 × 2), for the 2 × 2 block, there are 16 distinct configurations corresponding to different combinations of 0 and 1 bits. The absorption results of 5 cases (MB2 × 2–1 to MB2 × 2–5) are shown in Fig. 3(a) for the illustration and two best selective cases (in bold curves) are chosen for the combinational process to generate the full-sized structure. The corresponding two best configurations (MB2 × 2–1 and MB2 × 2–2) are also illustrated in the inset of Fig. 3(a). In the same scenario, the simulation results of different types of MB as MB3 × 3, MB4 × 4, and MB6 × 6 are presented in Fig. 3(b–d). For each type of MB, we choose two best configurations (which own two best absorption curves in bold) to combine the full-sized structure. E.g. for MB3 × 3 type, MB3 × 3–1 and MB3 × 3–2 are chosen for the combination of a full-sized structure.

From the coding metamaterial perspective, a meta-block could be treated as a ‘super-bit’, where the bit 0 or 1 is named by two best configurations of MB, for example, MB2 × 2–1 is set as bit 2–1 (for ON state) and MB2 × 2–2 is set as bit 2–0 (for OFF state), that is why in the insets of Fig. 3, we set the name ‘bit MB’ for the configurations. By choosing the suitable ON/OFF state of this super-bit on a surface of 144 UCs, we can optimize the structure to the widest absorption band by combining the MB or bits in the most convenient position. This is also the way that our algorithm performs in the simulation process to combine MBs (or ‘super-bit’) to find the optimal full-sized absorber structure. According to the same principle, we call the MB3 × 3–1 and MB3 × 3–2 are bit 3–1 and 3–0; MB4 × 4–1 and MB4 × 4–2 are bit 4–1 and 4–0; MB6 × 6–1 and MB6 × 6–2 are bit 6–1 and 6–0, respectively. These bit digits are illustrated in the inset of Fig. 3. When applied in practice, by integrating the ON/OFF circuit into the location of each MB, we can easily implement the desired full-sized configurations through a designed digital controller.

**Figure 4:**
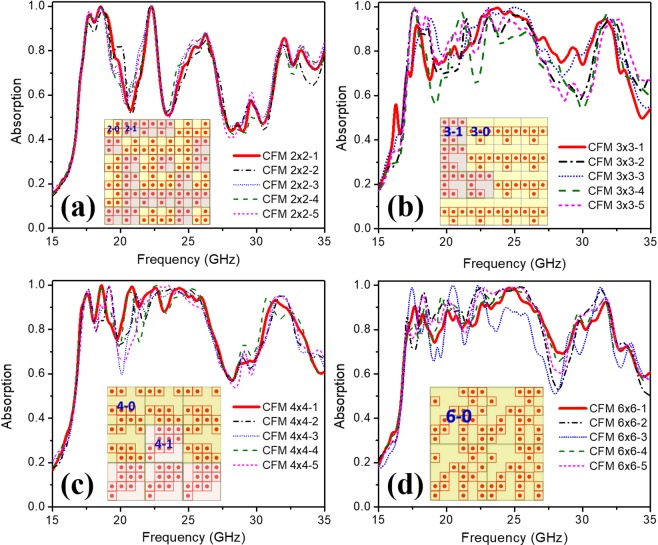
Absorption spectrum of full-sized absorbers after the combination of: (**a**) MB2 × 2 type, (**b**) MB3 × 3 type, (**c**) MB4 × 4 type and (**d**) MB6 × 6 type. The two types of colors in each full-sized configuration illustrate two different types of bits.

In the next step, each type of MB will be used to combine the full-sized configuration of 144 UCs. With MB2 × 2 type, 36 MBs are employed to combine the full-sized structure. The number of possibilities of simulations for this type is 2^36^. As an illustration of a straightforward approach and similar to the discussion in the SRS method, we calculate the absorption spectrum for a number of different structures, i.e. 200 structures and the absorptions of 5 selective cases are shown in Fig. 4(a). The best full-sized configuration, in this case, is assigned as CFM2 × 2–1 and is illustrated in the inset of Fig. 4(a). We see that this combination process of MB2 × 2–1 and MB2 × 2–2 does not greatly improve the absorption of the structure.

With MB3 × 3 type, we need 16 MBs to combine the full-sized structure of 144 UCs. The number of investigated simulation is 2^16^. As in the previous case, we perform 200 simulation rounds and 5 selective cases are shown in Fig. 4(b). The MB3 × 3-based full-sized structures show that there exists an optimal configuration with a superior result with the average absorptivity in the frequency range of 16–33 GHz is over 90% and the maximal absorption reaches 98%, this full-sized configuration is assigned as CFM3 × 3–1 and is illustrated in the inset of Fig. 4(b). In this case, the absorption of the full-sized configuration has been significantly improved compared to the SRS method.

The scenario is similar for the combination using the MB4 × 4 and MB6 × 6. The structure of 4 × 4 type needs 9 MBs then 2^9^ simulations could be performed. The 6 × 6 type requires 4 MBs so only 2^4^ simulations are needed for the investigation. The simulations are then performed and the absorption of five selective cases of each type is reported in Fig. 4(c,d). The inset of Fig. 4(c,d) also shows two best full-sized configurations CFM4 × 4–1 and CFM6 × 6–1, respectively.

Thus, the structures show that they can be optimized for a wide absorption frequency range from 16–33 GHz with all case of MB, but the MB3 × 3 types (configuration CFM3 × 3–1) shows the most effective improvement of the CFM method compared to the SRS method. A more detailed description of the simulation method and additional results can be found in Supporting Information.

## Field and Current Distribution in the Structure

**Figure 5:**
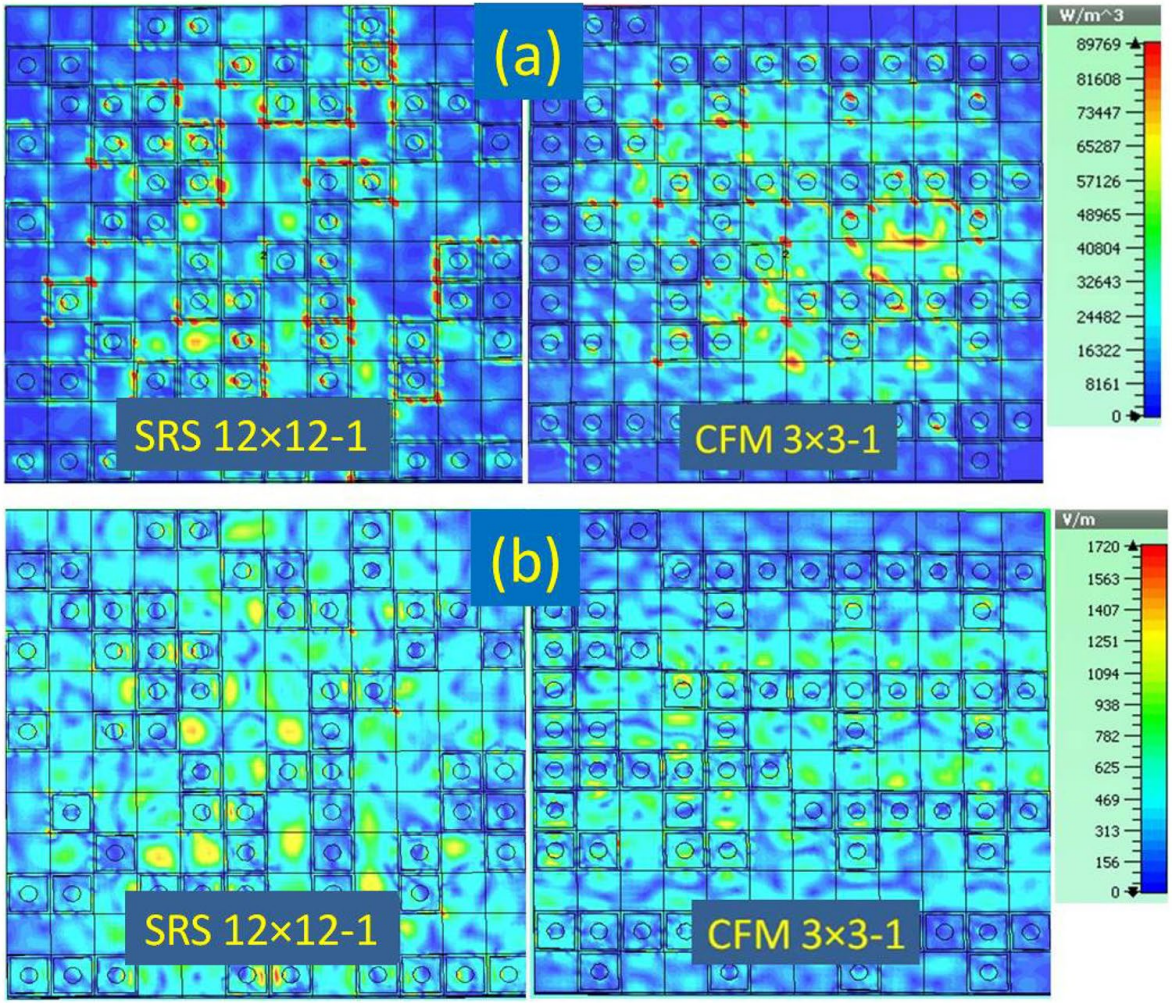
(**a**) The simulated power loss energy density on SRS and CFM structures at 25 GHz, which is in the absorption frequency range of the full-sized absorbers; (**b**) The simulated electric field density on SRS and CFM structures at 25 GHz.

To clarify the mechanism of energy absorption in the structure, the power loss density distribution is explored through simulation at the frequency of 25 GHz (inside the absorption band) for two full-sized configurations SRS12 × 12–1 and CFM3 × 3–1. As illustrated in Fig. 5(a), interestingly, one can see that almost the energy is concentrated in the coding defect region in the absorption band (at 25 GHz). This shows that when heat locally generated in the structure, it can be radiated quickly into the environment without harming devices using absorber.

The electric field distribution of SRS12 × 12–1 and CFM3 × 3–1 structures at 25 GHz is also provided to further clarify the mechanism of the absorption (Fig. 5(b)). Similar to the case of the power loss density, it is easy to point out that the electric field density is highly focused on the coding defect region.

**Figure 6:**
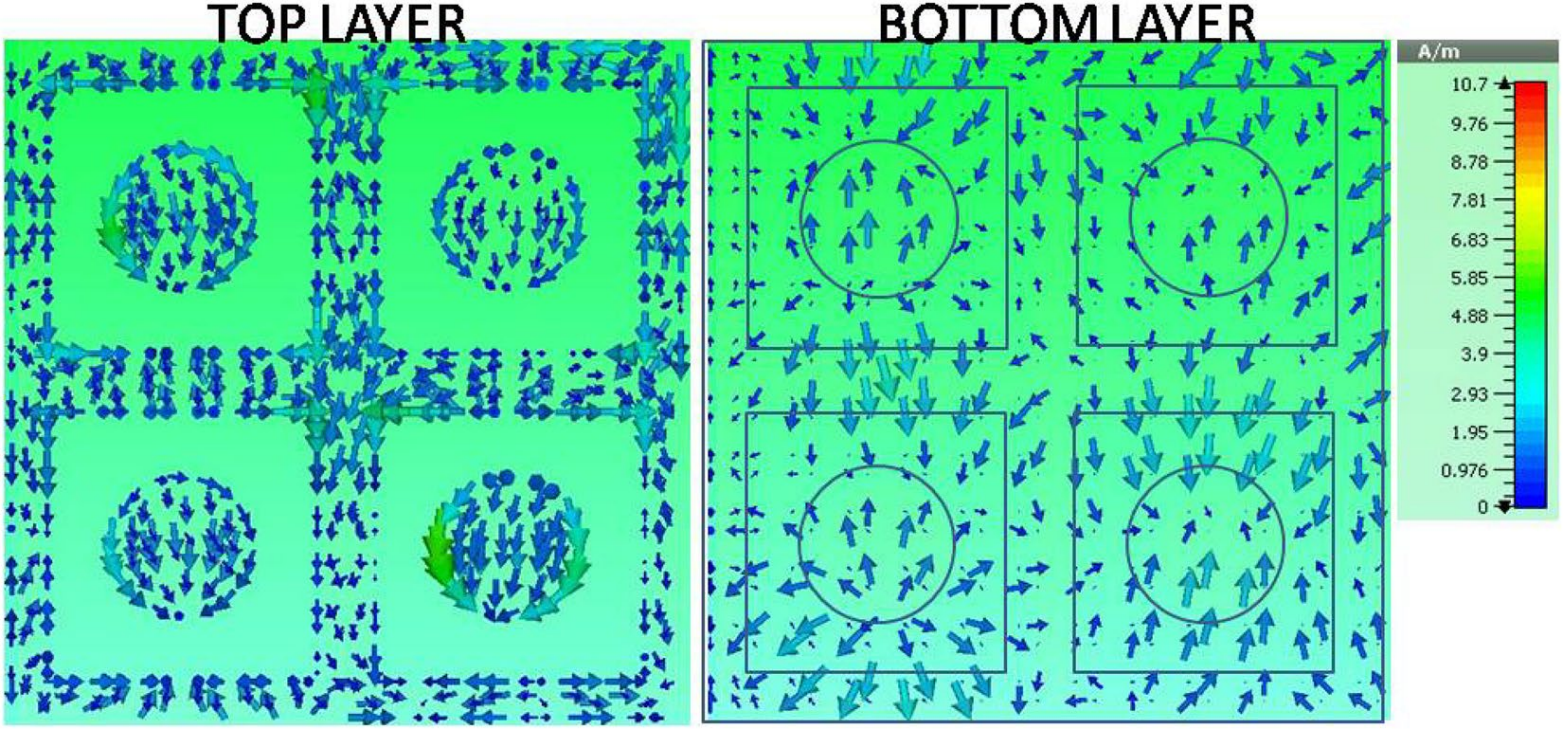
Simulation of the surface current of the unit cells at the absorption frequency of CFM3 × 3–1.

Figure 6 shows the surface current density of the structure for a set of four basic UCs from the configuration CFM3 × 3–1, the result indicates that the current density at the front and back layers of the structure is opposite. Observations at other UC positions at the absorption frequencies also provide similar results. This enables to form a magnetic resonance at the investigated absorption frequency.

## Absorption Mechanism Interpretation Using Equivalent Medium Theory (EMT)

**Figure 7:**
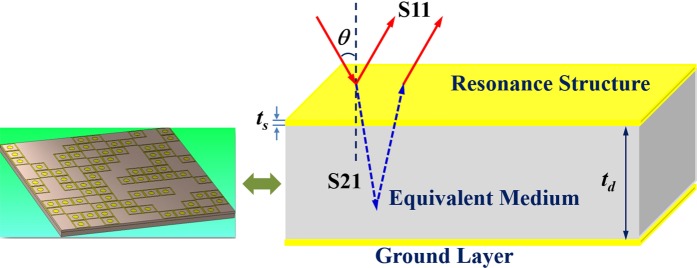
EMT model used for the calculation of the theory absorption.

To explain more clearly the mechanism of perfect absorption of the structure, we use the EMT (Equivalent Medium Theory) model (Fig. 7). This method can be seen in detail in our previous report^30^. Full 144 UCs structure, two most optimal configurations SRS12 × 12–1 and CFM3 × 3–1 which respectively generate by two approaches (SRS and CFM) are chosen for the investigation by EMT method. The EMT model considers the full-sized structure as an electromagnetic equivalent medium and is used to calculate effective impedance and extract the electromagnetic absorption of the structure from the total reflectance coefficient^29,30^.

**Figure 8:**
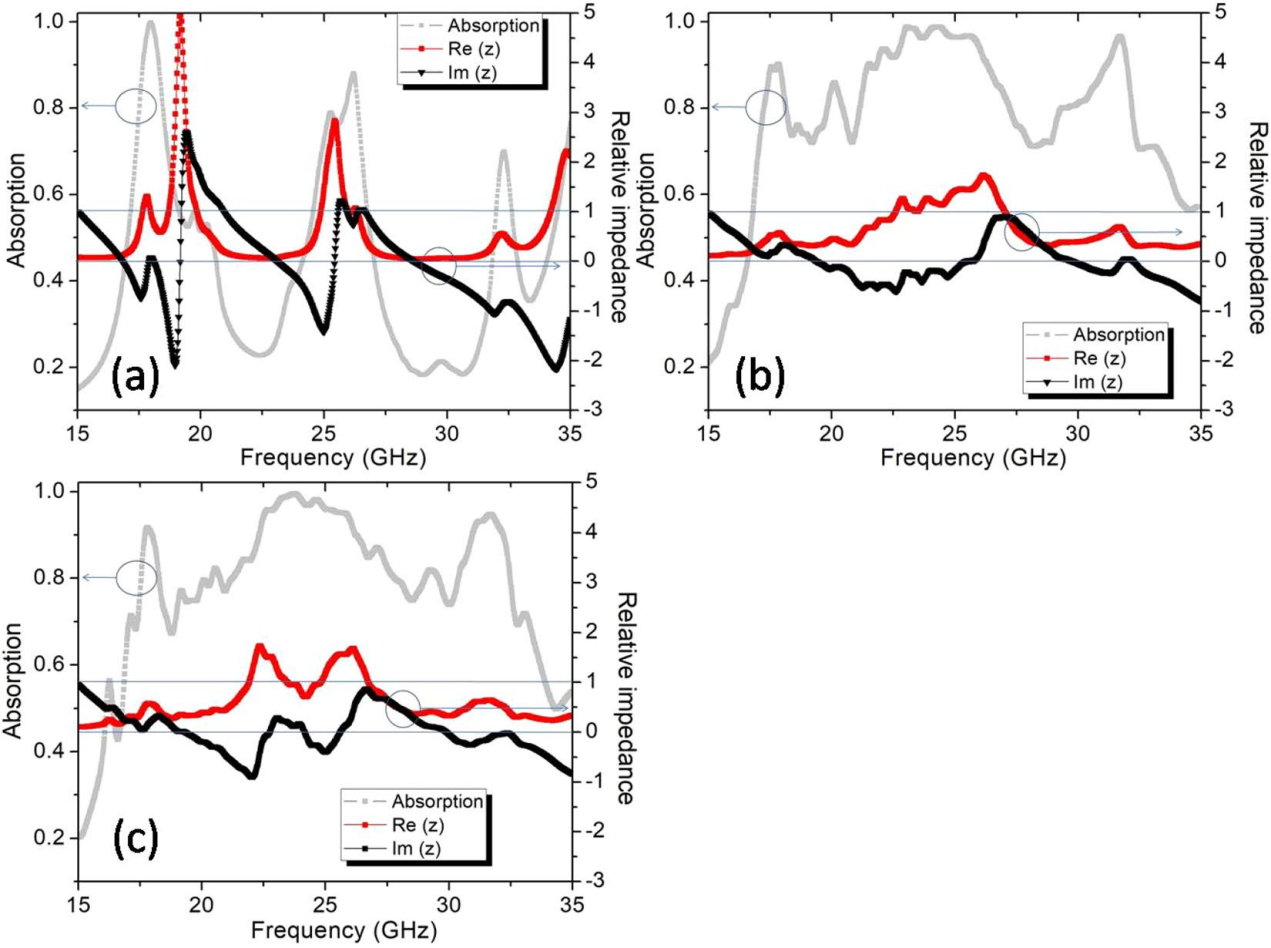
Relative impedance of the full-sized absorber types: (**a**) Full 144 UCs, (**b**) SRS12 × 12–1 and (**c**) CFM3 × 3–1.

In the absorption frequency range, the relative wave impedance z(ω) of the structure perfectly matches the environment and for the normal incidence, it is retrieved from the simulated S parameters^24,25^. The results of the extracted relative impedance for three configurations: Full 144 UCs structure, SRS12 × 12–1, and CFM3 × 3–1 are shown in Fig. 8 below. The real part of the relative impedance reaches unity (Re(z) ≈ 1) and the imaginary part is near to 0 (Im(z) ≈ 0), confirm that the impedance is matched well with the impedance of the free space in a range from 17 to 33 GHz. This provides a perfect absorption range for the structure and conducts the electromagnetic energy to be confined in the absorber.

**Figure 9:**
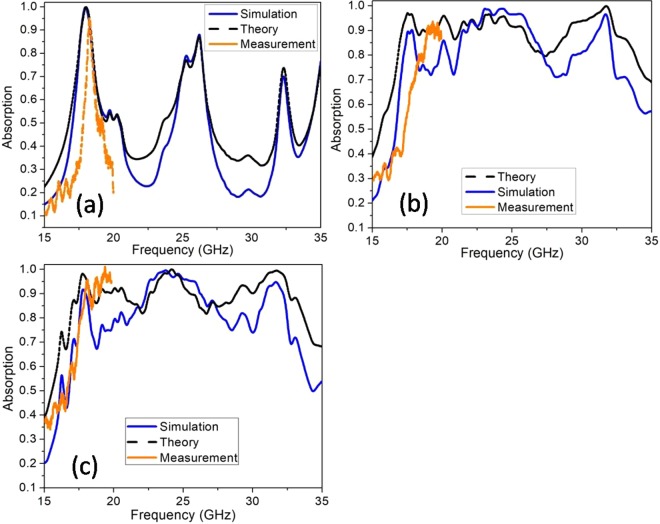
Comparison the simulation, theory and measured absorption spectrum of (**a**) full 144 UCs structure, (**b**) SRS12 × 12–1 structure and (**c**) CFM3 × 3–1 structure.

The calculation results of the absorption by EMT are shown in Fig. 9(a–c) below, the comparison with the simulation also presented in the same figure. The results are in good consistency.

## Experiment Verification

**Figure 10:**
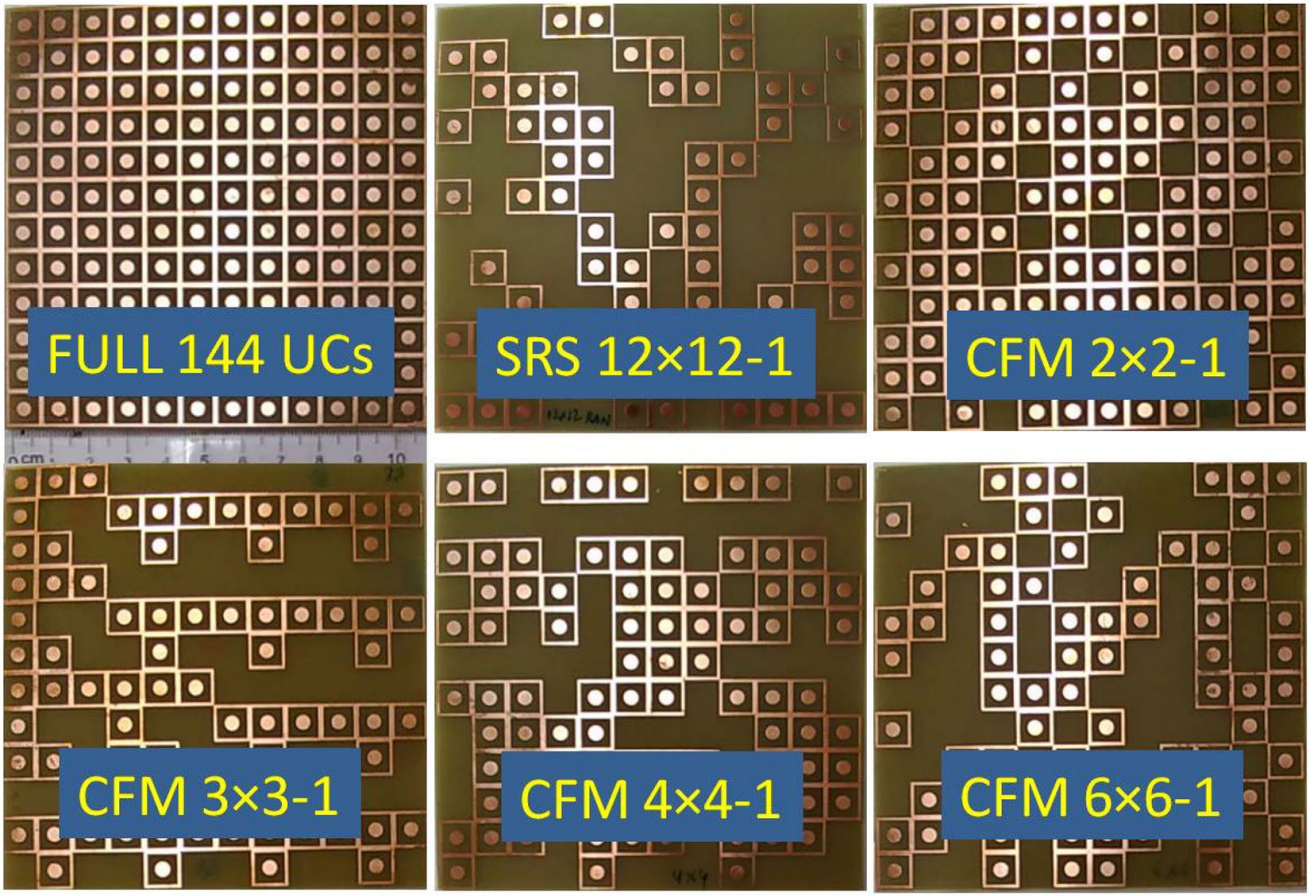
Image of the fabricated samples using for the measurement.

After the simulation, a full-sized non-defect structure, an optimized full-sized structure by SRS method (SRS 12 × 12–1), four most optimal absorber structures by CFM method (CFM 2 × 2–1, CFM 3 × 3–1, CFM 4 × 4–1, CFM 6 × 6–1) are fabricated and measured the absorption (Fig. 10). All samples have a size of 12 × 12 UC equivalent to an area of ~11 cm^2^. In the measurement, a vector network analyzer (Rohde & Schwarz ZNB20) with the working frequency range from 100 kHz to 20 GHz is used. Two standard horn antennas working at the same frequency range are applied for the electromagnetic emission and reception. The absorption rate is deduced from the reflection coefficient. Due to the limitation of our equipment, we perform the measurement only in the range of 13–20 GHz and the comparisons are also in this frequency range. It is clear that from 15 to 20 GHz, the measured results are in good agreement with the simulated ones for all cases (Fig. 9). From the tendency of the experimental results, we believe that the measurements will adapt to the simulations if equipment with a wider measuring range is applied. This also confirms the correctness of our method.

**Figure 11:**
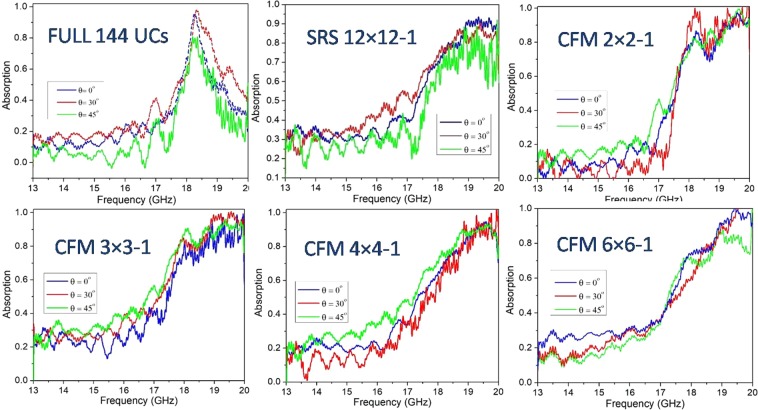
Measured angular dependence of the absorption of different full-sized absorbers under incident electromagnetic waves.

Additionally, the measured absorption spectra for different incident electric field polarization angles of all prototypes are shown in the frequency range from 13–20 GHz. As seen in Fig. 11, the absorption spectra of SRS and CFM structure depend weakly on the polarization of the incident wave until 45 degrees.

## Conclusions

This report presents a new study on broadband metamaterial absorber based on defect optimization coding of the full-sized metamaterial structure. Two approaches are presented for considering the broadband characteristic of the material. First, a full-sized structure is used to investigate the absorption when randomly removing the UC in the configuration. The second strategy uses the combination of meta-blocks of 2 × 2, 3 × 3, 4 × 4 and 6 × 6 UC to control and improve the absorption rate in the working frequency range. By integrating the digital concept into the metamaterial absorber system, namely coding metamaterials, the calculation, and analysis of the electromagnetic responses are significantly improved. A broadband absorber could be obtained in all cases when the optimal defect induced. The EMT model is then applied for understanding the absorption mechanism. Samples are fabricated for validating experimentally the proposed structures. The results show that the optimized absorbers work steadily in the 16–33 GHz range with high absorptivity up to 98%. The full-sized metamaterial absorbers designed have great potential in the community of anti-detector for the radar system, electromagnetic energy harvesting and so on. The presented method could pave a new road for the study and realization of the broadband coding metamaterial absorbers.

## Methods

### Simulation

Different configurations are generated using our computational algorithm which associated with the simulator. Each configuration corresponds to a two-dimensional logic frame of *n × n* unit cells where bit “0” represents the cell without metal plate, namely defect state, and bit “1” represents the cell with the metal plate, *n* is the number of row or column of the full-sized structure. Simulations are then performed using the commercial simulator CST Studio Suite. We use the open boundary conditions in the investigation process. A waveguide port is used for the excitation; the absorption is calculated from the magnitude of *S*_11_ parameters. All results are collected and analyzed afterward.

### Theory calculation

As in^30^, the final absorptivity is calculated through the total reflection (*R*_*tot*_) as A=1−*R*_*tot*_ while the total reflection is given from the real part and imaginary part of *S*_11_ and *S*_21_ (the transmission equals zero by using a metal plate at the back of the structure), which are extracted from the numerical simulation. Our Matlab code was used for data processing.

### Measurements

Measurements have been realized using a vector network analyzer R&S ZNB20 and a pair of standard horn antennas (emission and reception), working in the range of 100 kHz–20 GHz. The reflection coefficient is normalized using a full metal reflector at the same sample position. The angle of incidence wave is altered by changing the position of the horn antennas around the sample surface normal. The experimental results are compared with the simulations.

## Supplementary information

Supplementary Information.
