# Supplementary material for: Broadband microwave coding metamaterial absorbers
Source: Sci Rep. 2020 Feb 4;10:1810. doi: 10.1038/s41598-020-58774-1 (PMC7000747; doi:10.1038/s41598-020-58774-1)
Supplement: Supplementary file 1 — Supplementary Information. [file 41598_2020_58774_MOESM1_ESM.pdf]

SUPPORTING INFORMATION

Broadband microwave coding metamaterial absorbers

Manh Cuong Tran[1], Van Hai Pham[1], Tuan Hung Ho[1], Thi Thuy Nguyen[1], Hoang Tung Do[2], Xuan Khuyen Bui[3], Son Tung Bui[3], Dac Tuyen Le[4], The Linh Pham[3], Dinh Lam Vu[5]

[1]Faculty of Physics, Hanoi National University of Education, 136 Xuan Thuy, Cau Giay, Hanoi, Vietnam.
[2]Institute of Physics, Vietnam Academy of Science and Technology, Hanoi, Vietnam.
[3]Institute of Materials Science, Vietnam Academy of Science and Technology, 18 Hoang Quoc Viet, Hanoi, Vietnam.
[4]Department of Physics, Hanoi University of Mining and Geology, 18 Pho Vien, Bac Tu Liem, Hanoi, Vietnam.
[5]Graduate University of Science and Technology, Vietnam Academy of Science and Technology, 18 Hoang Quoc Viet, Hanoi, Vietnam.

*Email: tmcuong@hnue.edu.vn (M. C. Tran), haipv@hnue.edu.vn (V. H. Pham)

Simulation method

We represent each metamaterial structure as a 12×12 binary-matrix whose entries are either 0 and 1. The “1” bit is a unit cell including the outer square and inner disk; while without the square and disk the “0” bit (namely defect) is formed.

In a naive approach using a random binary matrix, the number of possible matrices would be so astronomically large ($\mathcal{O}(2^{12^2})$) that a numerical evaluation of all absorption spectra would be unfeasible. As an illustration of a straightforward approach, we calculate the absorption spectrum for a number of different structures, i.e. 200 structures- which is obviously much less than the number of possible structures. The numerical results for these structures are also presented in Figure S1 (Supporting Information) and shown below.

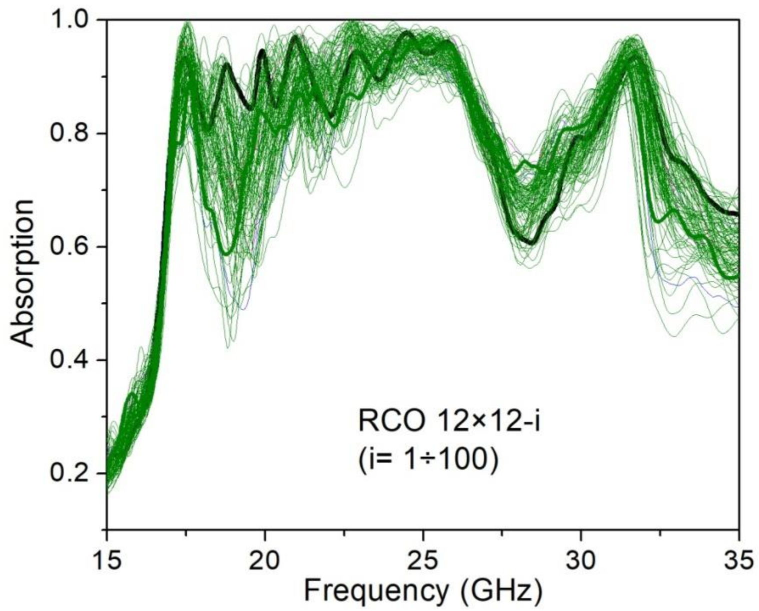

Figure S1: Absorption obtained using random configuration optimization (RCO) method for 12x12 unit cell (UC) type. Shown are only 100 different absorption curves (blue line). The bold curves belong to two of hundreds are for the observation purpose only.

Compared to a full-sized absorber without any defects (blue line), most of randomly generated structures with defect show a wider absorption band in the range 16 – 33 GHz. However, one can see that a large number of such structures suppress the absorption below 70% at around frequencies of 19 and 28.3 GHz.

To overcome this problem we propose another algorithm, which is based on a combination of fundamental meta-blocks. Here, each meta-block is actually a $p \times p$ random binary matrix, where $p = 2,3,4$ and 6. The reason for choosing these block species is that the number of possible structures, $\mathbb{N}(p)$, generated by a random binary matrix generator is not too large. For example, for $p = 2$, $\mathbb{N}(2) = 16$; $p = 3$, $\mathbb{N}(3) = 512$. Furthermore, it should be noted that the p values are the divisors of 12, the $p \times p$ matrix that is basically a submatrix of a 12×12 matrix, therefore the $p \times p$ matrix can be used as building blocks for a combination process in the following procedure.

1) A series of simulations were performed on 2×2 matrices to obtain a variety of electromagnetic responses. Mathematically, there are 16 possible structures corresponding to 16 coding sequences of the “0” bit and “1” bit for the 2×2 building block. However, in fact, we observed only 5 distinct absorption spectra (shown in Fig. 3a) because the equivalent configurations, e.g. 1000, 0100, 0010, 0001, give rise to the same absorption spectrum. Among the resulting structures, we chose two structures with the widest absorption range and the highest intensity to build up hierarchical superstructures. In particular, one of two chosen structures must possess a high absorption intensity (at least 90%) at frequencies of 19 and 28.3 GHz.

2) Using two structures obtained above called superbit “0” and “1”, respectively, we carried out simulations with 200 different structures as in the SRS method. Each structure was generated by a 6x6 random matrix of superbit variables. So, such a matrix is a 12x12 matrix of ordinary binary numbers. The results for 100 structures, labelled as CFM $2 \times 2 - i$ with $i = 1 \div 100$, are shown below.

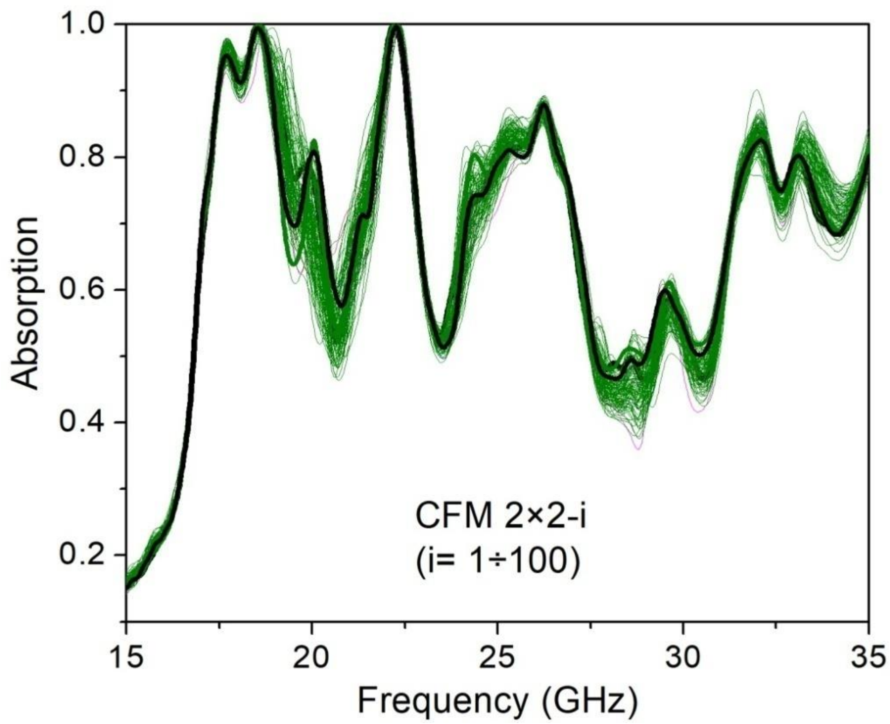

Figure S2: Absorption obtained using combination of fundamental meta-block (CFM) method based on a combination of two 2×2 meta-blocks. Shown are only 100 different absorption curves. The bold curves belong to two of hundreds are for the observation purpose only.

In these CFM structures, there typically exists weak absorption at several frequencies. Therefore, in order to improve the absorption efficiency, we next consider the 3×3, 4×4 and 6×6 meta block in the manner similar to the 2×2 meta block as described in the step (1) and (2).

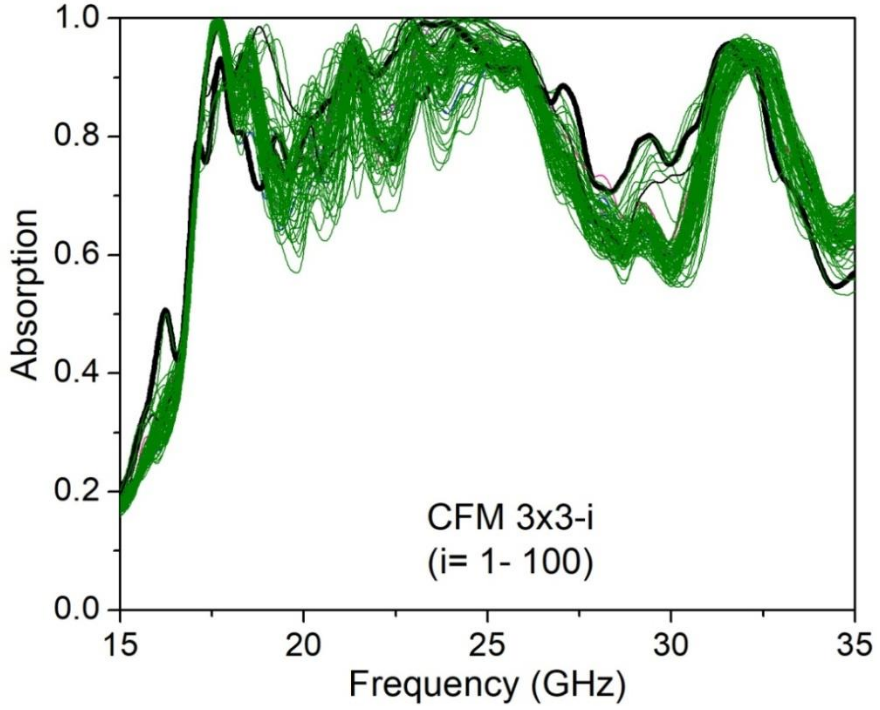

Figure S3: Same as Figure S2, but for 3×3 meta-blocks

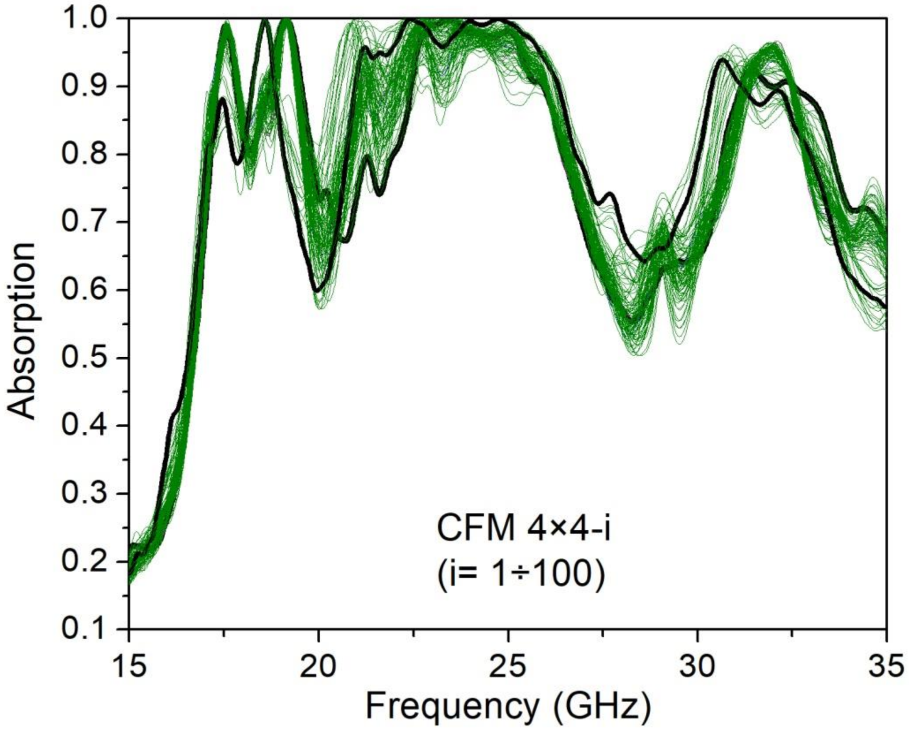

Figure S4: Same as Figure S2, but for 4×4 meta-blocks

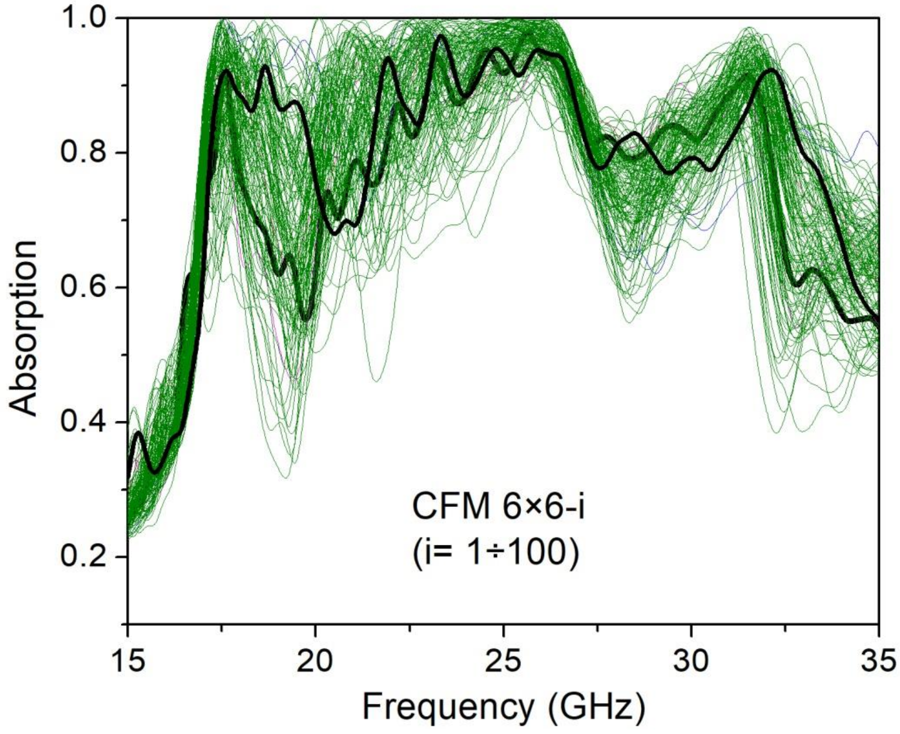

Figure S5: Same as Figure S2, but for 6×6 meta-blocks

Clearly, compared to the CFM 2×2 meta-block and RCO method, many structures of the CFM 3×3, 4×4 and 6×6 meta-blocks show a high absorption in a broadband range. Moreover, since the number of distinct structures of these meta-blocks is on the scale of hundreds to thousands of simulations, which is easily feasible for the current simulation computer. This suggests that CFM 3×3 or 4×4 meta-block can be used to optimize hearchically organized structures. It should be also noted that our method also be applied to the full-sized structures with arbitrary dimensions, and thus provide a useful strategy in the design of metamaterials with specific desired frequency ranges.

Reflection coefficient

For the ease of observation the reflection coefficient for typical structures is shown below. One can see that in all cases the S11 parameters relating to the reflection coefficient by the equation, is below -10 dB in the range of interest. Based on these S11 parameters, we calculated the absorption by the equation $A(\omega) = 1 - |S11|^2$

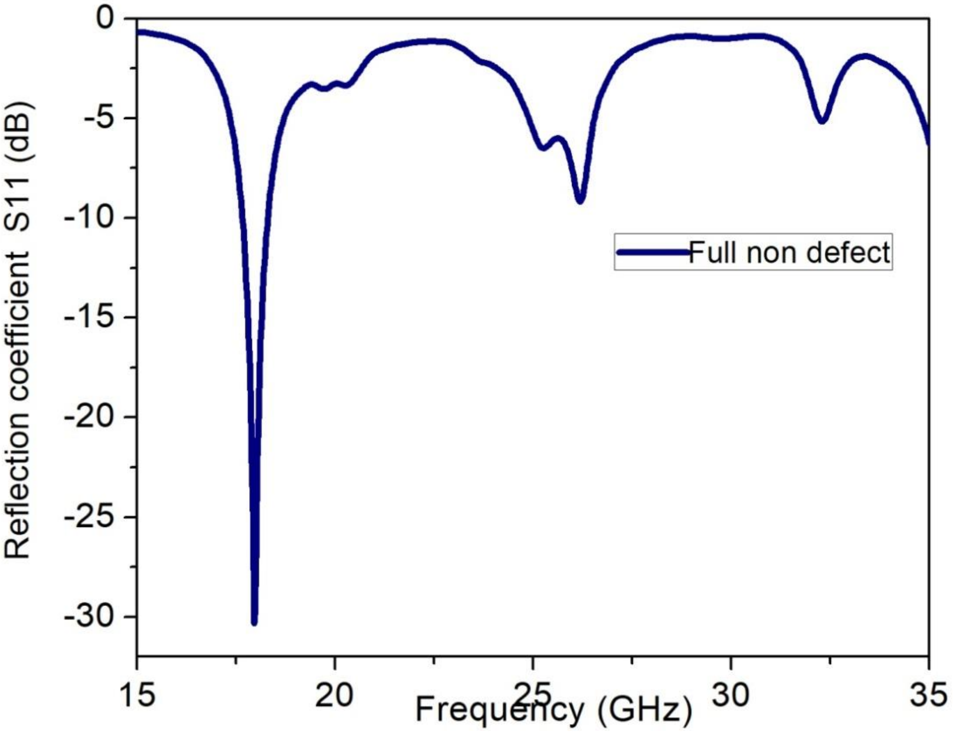

Reflection coefficient S11 (dB)
Frequency (GHz)
Full non defect

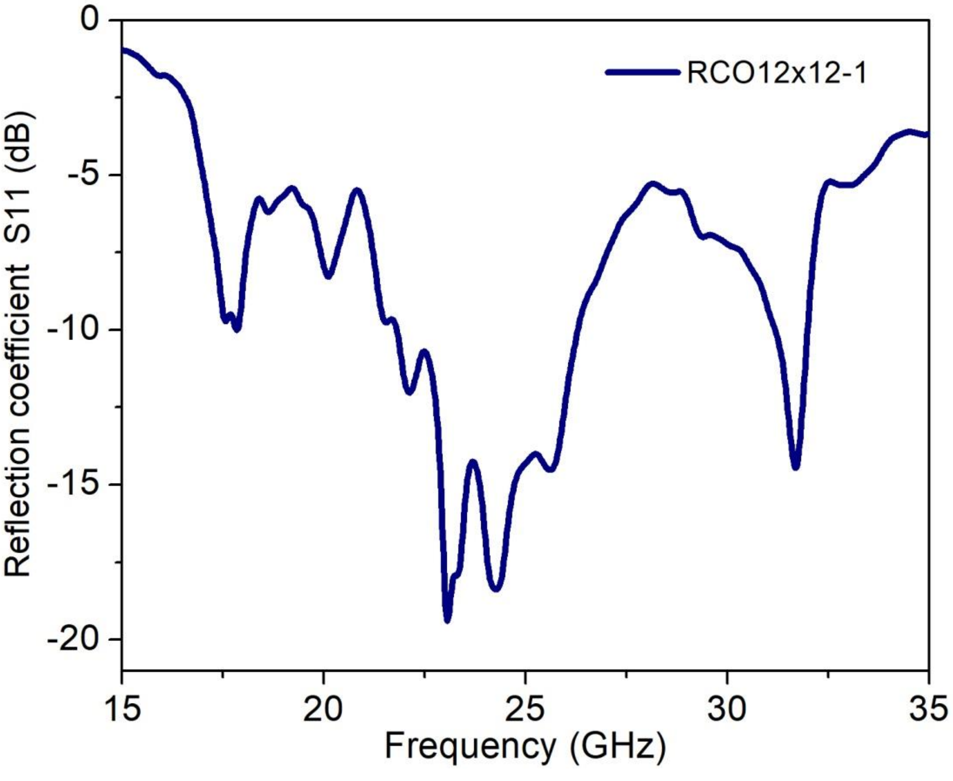

Reflection coefficient S11 (dB)
Frequency (GHz)
RCO12x12-1

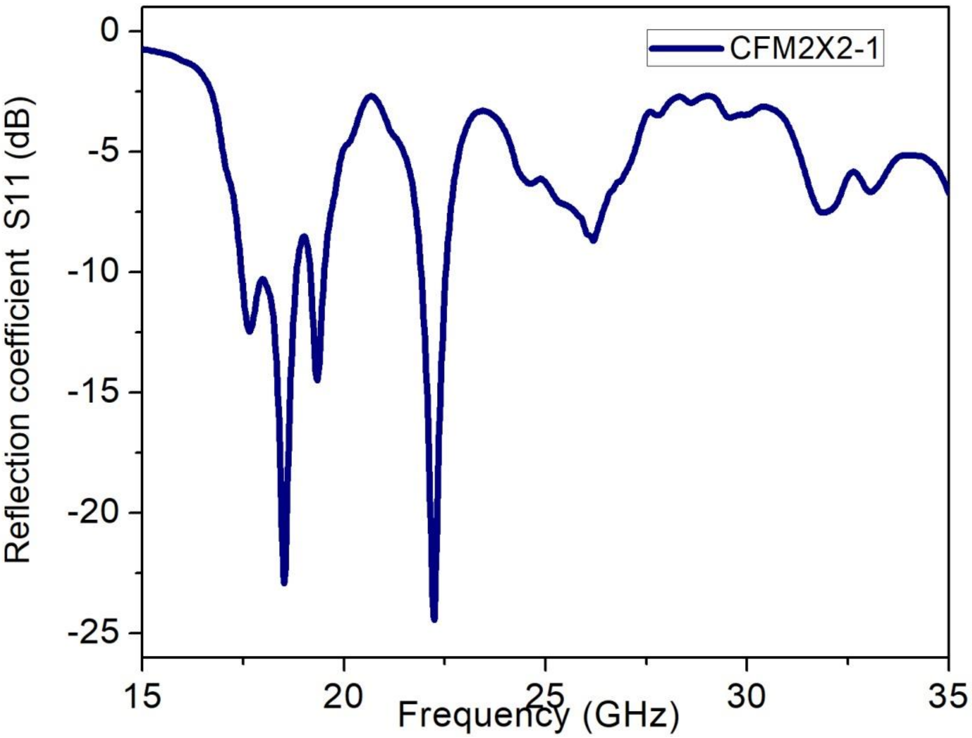

CFM2X2-1
Reflection coefficient S11 (dB)
Frequency (GHz)
0
-5
-10
-15
-20
-25
15
20
25
30
35

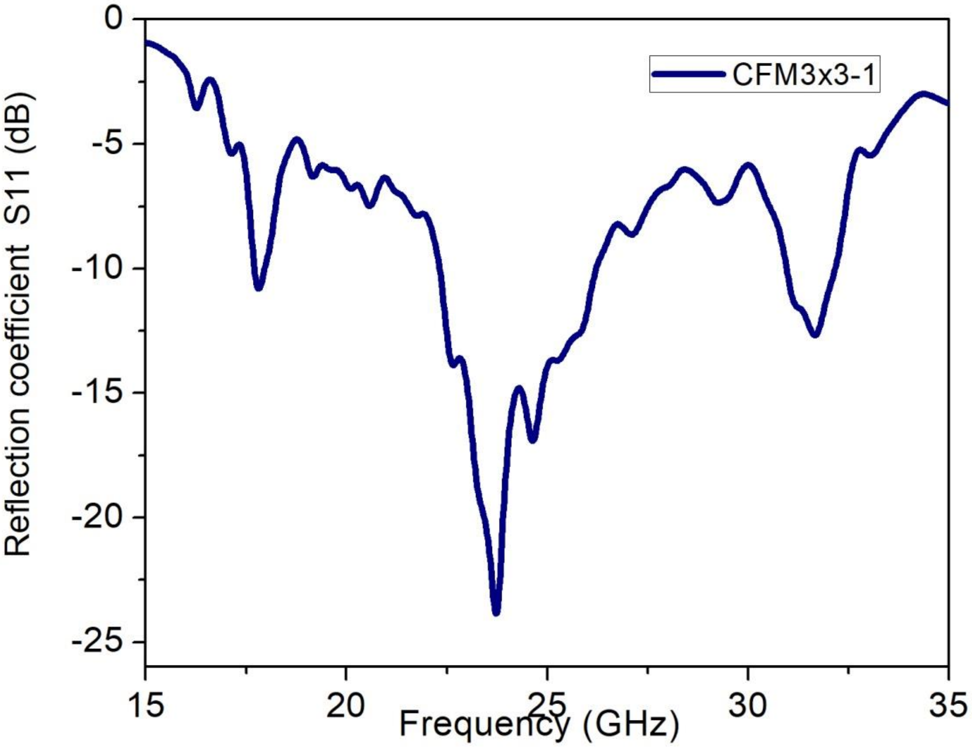

CFM3x3-1
Reflection coefficient S11 (dB)
Frequency (GHz)
0
-5
-10
-15
-20
-25
15
20
25
30
35

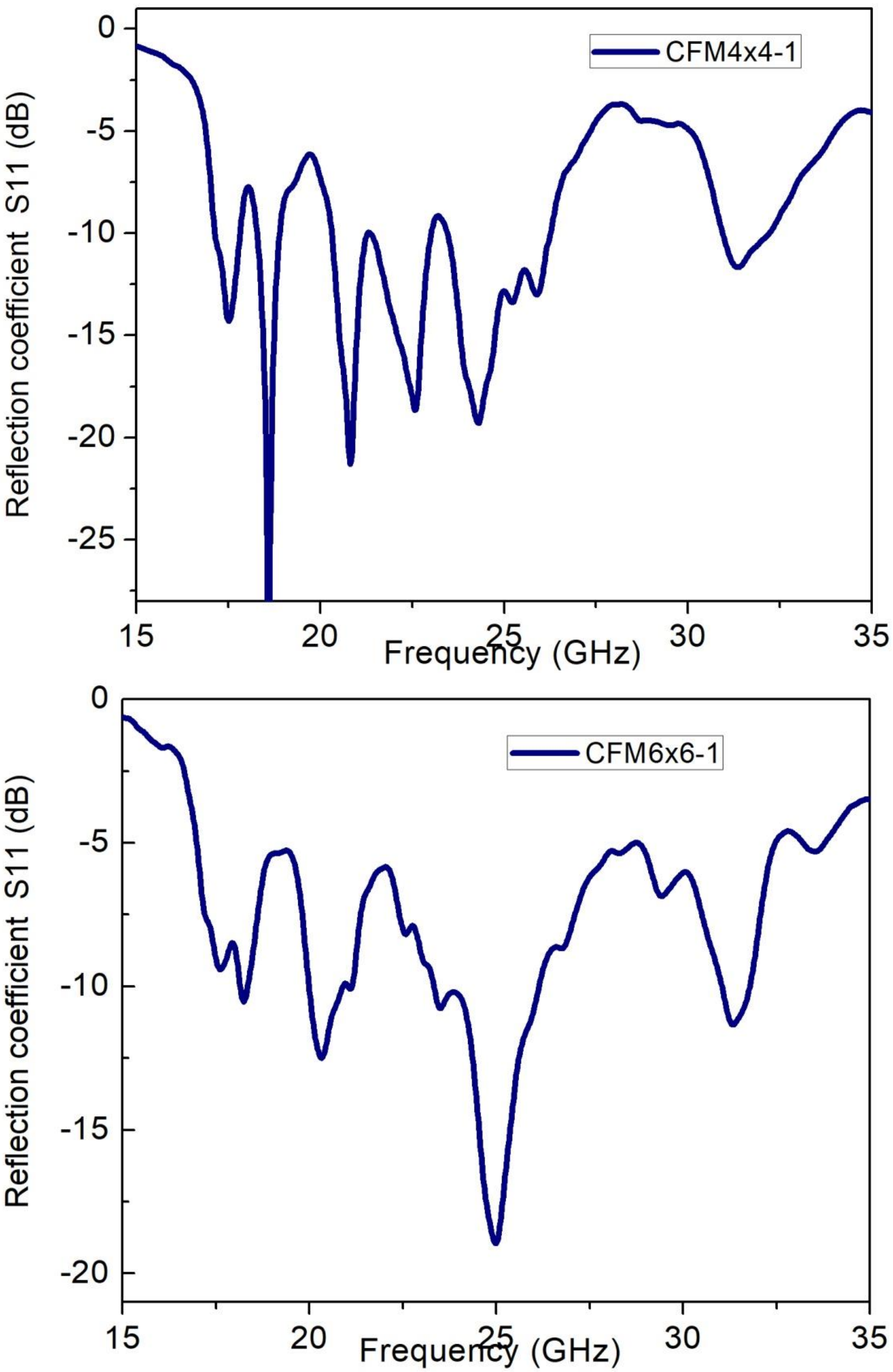

Figure S6: Reflection coefficients of the absorber strutures in the study (6 cases).
